# The genus *Paraplonobia* Wainstein and *Neopetrobia* Wainstein (Acari, Trombidiformes, Tetranychidae) from Saudi Arabia: new species, new records and key to the world species of *Paraplonobia*

**DOI:** 10.3897/zookeys.598.9060

**Published:** 2016-06-14

**Authors:** Muhammad Kamran, Jawwad Hassan Mirza, Fahad Jaber Alatawi

**Affiliations:** 1 Acarology Laboratory, Department of Plant Protection, College of Food and Agricultural Sciences, King Saud University, 11451, P.O. Box 2460, Riyadh, Saudi Arabia

**Keywords:** Hystrichonychini, arabica, haloxylonia, tabukensis, Prosopis

## Abstract

The two tetranychid genera *Paraplonobia* Wainstein and *Neopetrobia* Wainstein (Trombidiformes: Tetranychidae) are reported for the first time from Saudi Arabia. Three new species Paraplonobia (Anaplonobia) arabica Mirza & Alatawi, **sp. n.**, Paraplonobia (Anaplonobia) haloxylonia Alatawi & Mirza, **sp. n.** and Paraplonobia (Anaplonobia) tabukensis Kamran & Alatawi, **sp. n.** are described and illustrated based on adult females, collected from *Prosopis juliflora* (SW.) Dc. (Fabaceae) and *Haloxylon salicornicum* Bunge (Amaranthaceae) from two different regions of Saudi Arabia. *Neopetrobia mcgregori* (Pritchard and Baker) is redescribed and illustrated based on female collected from *Cynodon dactylon* L. (Poaceae).The diagnostic morphological features including leg chaetotaxy of all known species of the subgenus *Anaplonobia* is tabulated. A key to the world species of the genus *Paraplonobia* is also provided.

## Introduction

The genus *Paraplonobia* Wainstein belongs to the tribe Hystrichonychini Pritchard and Baker of the subfamily Bryobiinae (Acari: Prostigmata: Tetranychidae). Wainstein (1960) considered *Anaplonobia* and *Paraplonobia* as subgenera of *Aplonobia* Womersley. Later, Tuttle and Baker (1968) proposed *Anaplonobia* and *Paraplonobia* as two valid genera. After that, Gutierrez (1985) categorized the genus *Paraplonobia* into three subgenera: *Anaplonobia* Wainstein, *Brachynychus* Mitrofanov & Strunkova and *Paraplonobia* Wainstein on the basis of coxal setal count and the aspect of peritremes and considered the genus *Anaplonobia* as subgenus of *Paraplonobia* (Gutierrez 1985).

The genus *Paraplonobia* includes 32 species to date, which are widely distributed throughout the world. The subgenera *Anaplonobia*, *Paraplonobia*, and *Brachynychus* include 22, nine and one species, respectively (Baker and Tuttle 1972, Meyer 1987, Bolland et al. 1998, Migeon and Flechtmann 2004).

The subgenera *Anaplonobia* and *Paraplonobia* have a coxal setal formula of 2–2–1–1 while the subgenus *Brachynychus* has a coxal setal formula of 4–3–2–2. The subgenus *Anaplonobia* differs from *Paraplonobia* by having anastomosed peritremes while the later has simple peritremes (Gutierrez 1985, Bolland et al. 1998).

The genus *Neopetrobia* also belongs to the tribe Hystrichonychini and morphologically closely resembles the genus *Paraplonobia* except for the fourth pair of dorsocentral setae f_1_ which are widely spaced as compared to setae c_1,_ while f_1_ setae are normally spaced as c_1_ in *Paraplonobia* (Meyer 1987, Bolland et al. 1998). The genus *Neopetrobia* has been categorized into three subgenera; *Neopetrobia*, *Reckia* Wainstein and *Langella* Wainstein (Gutierrez 1985, Bolland et al. 1998). The subgenus *Neopetrobia* is different from other two subgenera by having integument without tuberculate or reticulate pattern and rounded or spindle shaped dorsal setae and includes ten species to date (Bolland et al. 1998).

A few tetranychid species have been reported from Saudi Arabia (SA): *Bryobia praetiosa* Koch, *Eotetranychus fallugiae* Tuttle & Baker, *Eutetranychus orientalis* (Klein), *Eutetranychus palmatus* Attiah, *Oligonychus afrasciaticus* (McGregor), *Oligonychus pratensis* (Banks), *Tetranychus cinnabarinus* (Boisduval), *Tetranychus turkrestzni* (Ugarov & Nikolskii), and *Tetranychus urticae* (Koch) (Martin 1972, Alatawi 2011). The genus *Paraplonobia* is poorly known from Arabian peninsula. Previously, two species Paraplonobia (Anaplonobia) harteni Meyer and Paraplonobia (Paraplonobia) dactyloni Smiley & Baker were reported from Yemen (Meyer 1996; Smiley and Baker 1995).

Two genera, *Paraplonobia* and *Neopetrobia*, are reported upon for the first time from Saudi Arabia with three new species: Paraplonobia (Anaplonobia) arabica sp. n., Paraplonobia (Anaplonobia) haloxylonia sp. n. and Paraplonobia (Anaplonobia) tabukensis sp. n. which are described and illustrated based on adult females. The male of Paraplonobia (Anaplonobia) haloxylonia sp. n. is also described and illustrated. *Neopetrobia mcgregori* (Pritchard & Baker) is redescribed and illustrated based on female.

Diagnostic features of all known species of the subgenus *Anaplonobia* are provided including body morphological features, leg I length in comparison to body length, and leg chaetotaxy (Tables 1 and 2) as well as a key to the world species of the genus *Paraplonobia*.

**Table 1.** Some morphological diagnostic features of the world species of the subgenus *Anaplonobia*, genus *Paraplonobia*.

Species	Distribution	Dorsal setae		Dorsal Striations		Stylophore anteriorly	Peritremes
		Shape	Distance of dorso-central hysterosomal setae especially (c1, d1, e1) to the setae next in line	Hysterosoma medialy	Propodosomal shield		
^3^ Paraplonobia (Anaplonobia) acharis (Pritchard & Baker, 1955)	USA	slightly lanceolate	distinctly shorter	widely spaced transverse	dashed, weak, longitudinal	rounded	weak with two irregular branches
^7^ Paraplonobia (Anaplonobia) algarrobicola (Gonzalez, 1977)	Chile	subspatulate, on tubercles	distinctly shorter	widely spaced transverse	longitudinal	rounded	anastomose
^3^ Paraplonobia (Anaplonobia) ambrosiae (Tuttle, Baker & Abbatiello, 1976)	Mexico, USA	slender/ setiform	distinctly shorter	widely spaced transverse	tuberculate longitudinal	-	ball like anastomose
^7^ Paraplonobia (Anaplonobia) arabica Mirza & Alatawi, sp. n.	Jazan, Riyadh, Tabuk	subspatulate, e-f on small tubercules	distinctly shorter	widely spaced transverse	weak, irregular, longitudinal	slightly incised	elongate anastomose
^9^ Paraplonobia (Anaplonobia) boutelouae Tuttle & Baker, 1968	USA	subspatulate	distinctly shorter	closely spaced transverse	-	-	anastomose
^3^ Paraplonobia (Anaplonobia) brickellia Baker & Tuttle, 1972	USA	subspatulate	distinctly shorter	widely spaced transverse	strong tubercules/lobes	round, tapering distally	strongly rounded, anastomose
^7^ Paraplonobia (Anaplonobia) candicans (Meyer, 1974)	South Africa	subspatulate, on tubercles	distinctly shorter	widely spaced transverse	medially wide spaced longitudinal, remaining dashed	slightly incised	complex anastomose
^9^ Paraplonobia (Anaplonobia) concolor Chaudhri, Akbar & Rasool, 1974	Pakistan	lanceolate	distinctly shorter	closely spaced transverse	weak transverse	deeply incised	anastomose
^9^ Paraplonobia (Anaplonobia) contiguus (Chaudhri, Akbar & Rasool, 1974)	Pakistan	lanceolate	distinctly shorter	closely spaced transverse	dotted longitudinal	slightly incised	weak branched anastomose
^9^ Paraplonobia (Anaplonobia) daryaensis Chaudhri, Akbar & Rasool, 1974	Pakistan	lanceolate	distinctly shorter	closely spaced transverse	irregular, weak, longitudinal, medially transverse	slightly incised	anastomose
^9^ Paraplonobia (Anaplonobia) glebulenta (Meyer, 1974)	South Africa	lanceolate	distinctly shorter	Small tubercles making pattern		round	sausage anastomose
^2,9^ Paraplonobia (Anaplonobia) haloxylonia Alatawi & Mirza, sp. n.	Riyadh, KSA	lanceolate	distinctly shorter	closely spaced transverse	weak, longitudinal	slightly incised	weak anastomose with few long thread like branches
Paraplonobia (Anaplonobia) harteni (Meyer, 1996)	Yemen	lanceolate	distinctly shorter	closely spaced transverse	dashed, transverse	slightly incised	weakly anastomose with few branches
^1^ Paraplonobia (Anaplonobia) inornata (Meyer, 1987)	South Africa	slender /setiform	distinctly shorter	widely spaced transverse	coarse longitudinal, plate dashed	incised	weak branched anastomose
^7^ Paraplonobia (Anaplonobia) prosopis (Tuttle & Baker, 1964)	Miami USA, Marigat, Kenya	subspatulate	distinctly shorter	widely spaced transverse	longitudinal	-	anastomose
^2^ Paraplonobia (Anaplonobia) tabukensis Kamran & Alatawi, sp. n.	Tabuk, KSA	narowly lanceolate	distinctly shorter	closely spaced transverse	weak, longitudinal	rounded	small, compact, anastomose
^9^ Paraplonobia (Anaplonobia) theroni (Meyer, 1974)	South Africa	lanceolate, on tubercles	distinctly shorter	closely spaced transverse	dashed fine longitudinal	slightly incised	elongate anastomose
^8^ Paraplonobia (Anaplonobia) allionia Baker & Tuttle, 1972	USA	slender/ setiform	as long as/ slightly longer	closely spaced transverse	strong tuberculate longitudinal	rounded	small, elongate anastomose bulb
^3^ Paraplonobia (Anaplonobia) calame (Pritchard & Baker, 1955)	USA	slender/ setiform, on small tubercles	as long as/ slightly longer	widely spaced transverse	pebbled	rounded	three chambered branches
^5^ Paraplonobia (Anaplonobia) coldinae (Tuttle & Baker, 1964)	USA	slender/setiform	much longer	-	-	rounded	anastomose
^7^ Paraplonobia (Anaplonobia) juliflorae (Tuttle & Baker, 1968)	USA	subspatulate on small tubercles	longer	widely spaced tuberculate striate	tuberculate striate	rounded	Weak branched anastomose
^3^ Paraplonobia (Anaplonobia) artemisia Baker & Tuttle, 1972	Mexico, USA	slender, blunt distally	as long as/slightly longer	widely spaced tuberculate fold, tranverse	broken, irregular, longitudinal	rounded	elongate anastomose bulb
^4^ Paraplonobia (Anaplonobia) berberis Baker & Tuttle, 1972	USA	slender/setiform	as long as/ slightly longer	widely spaced broad folds with tubercules	small tubercules	rounded	small anastomose bulb
^6,7^ Paraplonobia (Anaplonobia) euphorbiae (Tuttle & Baker, 1964)	Mexico, USA	subspatulate	slightly shorter/as long as	irregular transverse	basket weave	rounded	anastomose
^9^ Paraplonobia (Anaplonobia) tshipensis (Meyer, 1974)	South Africa	spatulate, on tubercles	longer	closely spaced transverse	broken longitudinal	deeply incised	oval anastomose

Host Plant Family: 1. Acanthaceae, 2. Amaranthaceae, 3. Asteraceae, 4. Berberidaceae, 5. Boraginaceae, 6. Euphorbiaceae, 7. Fabaceae, 8. Nyctaginaceae, 9. Poaceae

**Table 2.** Length of leg I and leg chaetotaxy of world species of subgenus *Anaplonobia* genus *Paraplonobia*.

Species		Length of leg I as compared to body length	Coxa				Trochanter				Femora				Genua				Tibia				Tarsus			
			I	II	III	IV	I	II	III	IV	I	II	III	IV	I	II	III	IV	I	II	III	IV	I	II	III	IV
Paraplonobia (Anaplonobia) acharis		longer	-	-	-	-	-	-	-	-	-	-	-	-	-	-	-	-	13(1)	-	-	-	14(3)+2dup	-	-	-
Paraplonobia (Anaplonobia) algarrobicola		longer	2	2	0	0	1	1	1	1	5	5	3	3	4	4	3	2	10(2)	7	9	8	19	14	11	11
Paraplonobia (Anaplonobia) arabica	Tabook, Riyadh	longer	2	2	1	1	1	1	1	1	5	5	3	3	5	4	3	3	9(1)	9	9	9	14(2)+2dup	8(1)+1dup	8(1)	9(1)
	Jizan		2	2	1	1	1	1	1	1	5	5	3	3	5	4	3	3	9(1)	8	9	9	12(1)+2dup	9(1)+1dup	9(1)	9(1)
Paraplonobia (Anaplonobia) brickellia		longer	-	-	-	-	-	-	-	-	-	-	-	-	-	-	-	-	-	-	-	-	-	-	-	-
Paraplonobia (Anaplonobia) daryaensis		longer	2	2	1	1	1	1	1	1	9	6	4	4	5	5	4	4	14	9	9	9	22	15	15	15
Paraplonobia (Anaplonobia) haloxylonia		longer	2	2	1	1	1	1	1	1	9	6	4	4	5	5	4	4	13(1)	9	9	9	15(2)+2dup	10(1)+1dup	12(1)	12(1)
Paraplonobia (Anaplonobia) harteni		longer	2	2	1	1	1	1	1	1	9	6	4	4	5	5	4	4	13(1)	9	9	9	18(2)+2dup	12(2)+1dup	14(1)	14(1)
Paraplonobia (Anaplonobia) prosopis	Tuttle and Baker 1964	longer	-	-	-	-	-	-	-	-	-	-	-	-	-	-	-	-	11(1)	8	-	9	15(1)+2dup	9(1)+1dup	-	10(1)
	Toroitich et al. 2009		-	-	-	-	-	-	-	-	5	4	3	3	4	4	3	2	9	7	8	7	13(2)	10(1)	9	8
Paraplonobia (Anaplonobia) tabukensis		longer	2	2	1	1	1	1	1	1	8	6	3	3	4	5	3	3	13(1)	9	8	8	10+2dup	7+1dup	9+1dup	9+1dup
Paraplonobia (Anaplonobia) allionia		longer	-	-	-	-	-	-	-	-	-	-	-	-	-	-	-	-	-	-	-	-	-	-	-	-
Paraplonobia (Anaplonobia) artemisia		longer	-	-	-	-	1	-	-	-	6	-	-	-	4	-	-	-	9(1)	-	-	-	11(1)+2dup	-	-	-
Paraplonobia (Anaplonobia) berberis		longer	-	-	-	-	-		-	-	-	-	-	-	4	3	-	-	9(1)	5	-	6	8(1)+2dup	8	-	9
Paraplonobia (Anaplonobia) calame		longer	-	-	-	-	-	-	-	-	-	-	-	-	-	-	-	-	13(1)	-	-	-	13(2)+2dup	-	-	-
Paraplonobia (Anaplonobia) concolor		shorter	2	2	1	1	1	1	1	1	9	6	4	4	5	5	4	4	14	9	9	9	22	15	15	15
Paraplonobia (Anaplonobia) contiguus		shorter	2	2	1	1	1	1	1	1	9	6	4	4	4	5	4	4	14	9	9	9	20	15	15	15
Paraplonobia (Anaplonobia) tshipensis		shorter	-	-	-	-	-	-	-	-	9	6	4	4	5	5	4	4	13(1)	8	6	7	16(1)+2dup	13+1dup	12(1)	12(1)
Paraplonobia (Anaplonobia) ambrosiae		-	-	-	-	-	-	-	-	-	-	-	-	-	-	-	-	-	13(1)	-	-	-	16(3)+2dup	(1)+2dup	-	-
Paraplonobia (Anaplonobia) boutelouae		-	-	-	-	-	-	-	-	-	-	-	-	-	-	-	-	-	-	-	-	-	-	-	-	-
Paraplonobia (Anaplonobia) candicans		-	-	-	-	-	-	-	-	-	11	8	6	5	8-9	9	7	7	13(1)	9	9	9	18(1)+2dup	15+1dup	14(1)	14(1)
Paraplonobia (Anaplonobia) glebulenta		-	-	-	-	-	-	-	-	-	9	6	4	4	5	5	3	3	12-13(1)	9	9	9	16(2)+2dup	12-13(2)+1dup	12(1)	12(1)
Paraplonobia (Anaplonobia) innornata		-	2	2	1	1	1	1	1	1	9	6	4	4	5	5	6	6	13(1)	9	9	9	18(2)+2dup	14+1dup	14(1)	14(1)
Paraplonobia (Anaplonobia) theroni		-	-	-	-	-	-	-	-	-	9	6	4	4	5	5	4	4	13(1)	9	6	9	18(1)+2dup	14+1dup	12-13(1)	14(1)
Paraplonobia (Anaplonobia) coldinae		-	-	-	-	-	-	-	-	-	-	-	-	-	-	-	-	-	-	-	-	-	-	-	-	-
Paraplonobia (Anaplonobia) euphorbiae		-	-	-	-	-	-	-	-	-	-	-	-	-	-	-	-	-	-	-	-	-	-	-	-	-
Paraplonobia (Anaplonobia) juliflorae		-	-	-	-	-	-	-	-	-	-	-	-	-	-	-	-	-	-	-	-	-	-	-	-	-

## Materials and methods

The mite specimens were collected by shaking the plant parts, especially leaves, onto a white sheet of paper. Mites found moving on paper were collected with the help of a camel hairbrush and preserved in small vials containing 70% ethanol. Preserved mite specimens were observed under a stereomicroscope (SZX10, Olympus, Tokyo, Japan) and mounted on glass slides in Hoyer’s medium. The mounted specimens were examined under phase contrast microscope (DM2500, Leica, Wetzlar, Germany). Different body parts were pictured using an auto montage software system (Syncroscopy, Cambridge, UK), then drawn with Adobe Illustrator (Adobe System Inc., San Jose, CA, USA). All measurements are in micrometers. The terminology used in this paper follows that of Lindquist (1985). All type specimens were deposited at Acarology Laboratory, Department of Plant Protection, College of Food and Agricultural Sciences, King Saud University except one each of female and male paratypes of Paraplonobia (Anaplonobia) haloxylonia sp. n., female paratype each of Paraplonobia (Anaplonobia) arabica sp. n., and Paraplonobia (Anaplonobia) tabukensis sp. n., with Accession numbers, OSAL 0115769, OSAL 00115768, OSAL 0110333 and OSAL 0110332 respectively, that were deposited at Ohio State University Acarology Laboratory
(OSAL), USA.

## Results and discussion

### Family Tetranychidae Donnadieu Subfamily Bryobiinae Berlese

#### Genus Paraplonobia

Taxon classificationAnimaliaProstigmataTetranychidae

Wainstein, 1960

Aplonobia (Paraplonobia) Wainstein, 1960: 140.
Paraplonobia : Tuttle and Baker 1968: 48, Meyer 1974: 119, Chaudhri et al. 1974: 28, Gutierrez 1985: 75, Bolland et al. 1998: 7.

##### Type species.

Aplonobia (Paraplonobia) echinopsili Wainstein, 1960 by original designation.

##### Diagnosis.

Based on Baker and Tuttle 1968, Gutierrez 1955, Meyer 1974, Meyer 1987, Bolland et al. 1998.

Body oval; prodorsum without lobes and with three pairs of setae; dorsal opisthosomal setae ten pairs. Dorsal setae not set on prominent tubercles; setae f_1_ normal in position, coxal setal formula variable, most species with 2–2–1–1 except one species of the subgenus *Brachynychus* having 4–3–2–2 setae on coxae I–IV respectively; anal setae three pairs; peritremes simple/anastomosing; tarsus I with two sets of duplex setae, present close to distal end of tarsus; claws and empodium pad-like each with tenant hairs (Fig. 5A).

#### Subgenus Anaplonobia

Taxon classificationAnimaliaProstigmataTetranychidae

Wainstein

##### Diagnosis.

Based on Gutierrez 1985, Bolland et al. 1998.

Peritremes anastomosed, coxal setal formula 2–2–1–1.

The subgenus *Anaplonobia* includes 22 species (Migeon and Flechtmann 2004). The species of the subgenus *Anaplonobia* can be grouped into two categories: 1) Eight species with dorsal body setae slightly shorter/as long as or longer than distances to the bases of consecutive setae (Tables 1 and 2), second group with dorsal body setae distinctly shorter than distances between their bases, contains 17 species including three new species (Paraplonobia (Anaplonobia) arabica sp. n., Paraplonobia (Anaplonobia) haloxylonia sp. n., and Paraplonobia (Anaplonobia) tabukensis sp. n.) reported in this study (Table 1, 2).

Shape of setae (spatulate, subspatulate, lanceolate or setiform), comparative length of setae with respect to the distance of setae next behind, shape of peritremes (compact anastomose, branched or weakly anastomosed), propodosomal shield (pebbled, lobbed, with longitudinal/transverse striations), hysterosoma (medially with closely/widely spaced striations), comparative length of leg I with respect to body length (shorter/longer) and leg chaetotaxy are the major diagnostic characters vary among/within the species of subgenus *Anaplonobia* (Table 1, 2).

Most species of the subgenus *Anaplonobia* have been reported from USA, Mexico, South Africa and Pakistan and collected mostly from three host plants families Asteraceaea, Fabaceae and Poaceae (Bolland et al. 1998) (Table 1).

The specimens of new species Paraplonobia (Anaplonobia) arabica sp. n., collected from *Prosopis juliflora* from three different regions (Riyadh, Tabuk, and Jazan) of Saudi Arabia, are morphologically similar except for some variations in setal counts on Tibia II and Tarsus I–II–III. (Table 2). The variations in the setal count of leg I–II–IV (Tibia and Tarsus) in Paraplonobia (Anaplonobia) prosopis had been found also in the description made by Tuttle and Baker (1964) from USA and Toroitich and Ueckermann (2009) from Kenya (Table 2). However, in some other species of the subgenus *Anaplonobia*, setal variations on genua, tibiae and tarsi have been found among the different specimens collected from the same host and location within the same species. i.e. genua I (8–9) in Paraplonobia (Anaplonobia) candicans, tibia I (12–13) and tarsus II (12–13) of Paraplonobia (Anaplonobia) glebulanta, and tarsus III (12–13) of Paraplonobia (Anaplonobia) theroni (Table 2).

#### Paraplonobia (Anaplonobia) arabica sp. n.

Taxon classificationAnimaliaProstigmataTetranychidae

http://zoobank.org/200D2E10-9324-4C31-8B04-F08C8F33EBD1

Figs 1–2 , 3–8

##### Diagnosis.

Dorsal body setae subspatulate, serrate, expanded distally and distinctly shorter to the distances of setae next in line, first pair of dorsocentral setae c1 reaching 2/3 to the distance of setae d1, setae c1 almost 1.5 times widely spaced than setae f1, setae e_2_, f_1_, f_2_ and h_1_ set on small tubercles, dorsal hysterosomal striations widely spaced, propodosoma medially with longitudinal broken striations, stylophore with a small mediocephalic emargination, peritremes branched tube like compact anastomosing, leg I shorter than body length.

##### Description of holotype female

(n = 9). Measurement of holotype followed by 8 paratypes (in parenthesis) (Figs 1–8).

**Figures 1, 2.**
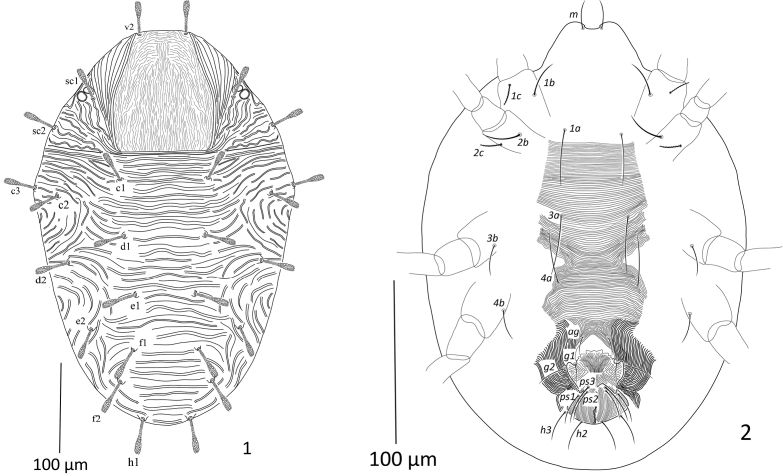
Paraplonobia (Anaplonobia) arabica sp. n. adult female. **1** dorsum **2** venter.

**Figures 3–8.**
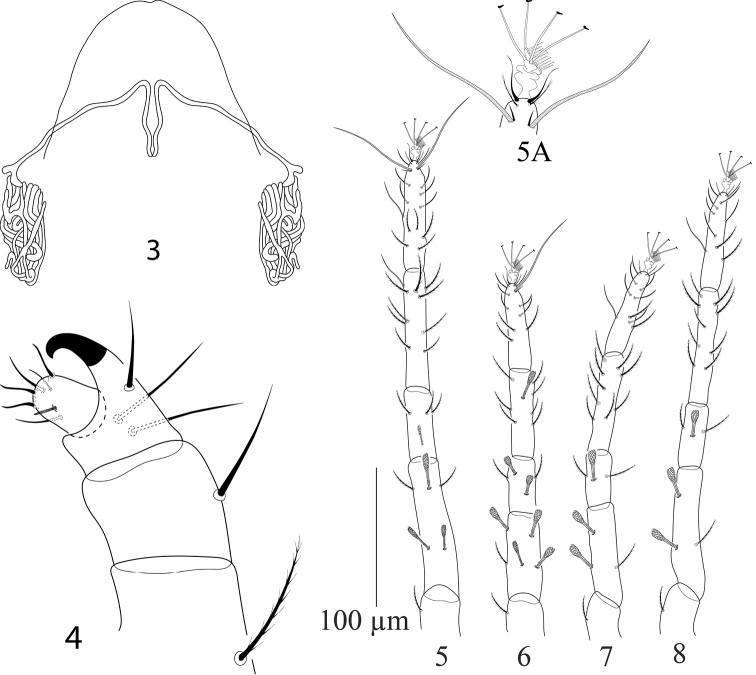
Paraplonobia (Anaplonobia) arabica sp. n. adult female. **3** stylophore and peritremes **4** palp **5** leg I **5A** duplex setae, empodium and claws of tarsus I **6** legII **7** legIII **8** leg IV.

**Dorsum** (Fig. 1). Body oval; length of idiosoma 439 (430–443), maximum width 282 (280–287), length of body (gnathosoma + idiosoma) 476 (472–480). Propodosoma medially with longitudinal broken striations, without anterior projections. Dorsal body setae subspatulate, serrate, expanded distally and distinctly shorter to the distances of setae next in line, first pair of dorsocentral setae c1 reaching 2/3 to the distance of setae d1, setae c1 almost 1.5 times widely spaced than setae f1, setae e_2_, f_1_, f_2_ and h_1_ set on small tubercles. Dorsal striations transverse on hysterosoma, without lobes and widely spaced. Length of dorsal setae: v_2_ 45 (42–46), sc_1_ 40 (38–41), sc_2_ 41 (40–43), c_1_ 45 (44–48), c_2_ 42 (40–44), c_3_ 40 (39–44), d_1_ 34 (32–38), d_2_ 44 (43–46), e_1_ 45 (44–48), e_2_ 44 (43–45), f_1_ 45 (44–45), f_2_ 44 (42–45), h_1_ 46 (45–48). Distance between dorsal setae: v_2_–v_2_ 53 (51–55), v_2_–sc_1_ 97 (95–98), sc_1_–sc_2_ 56 (54–57), sc_1_–sc_1_ 166 (162–167), sc_2_–sc_2_ 263 (260–266), c_1_–c_1_ 94 (92–96), c_1_–c_2_ 82 (80–85), c_2_–c_3_ 41 (39–44), c_2_–c_2_ 261 (260–264), c_3_–c_3_ 345 (340–346), d_1_–d_1_ 82 (80–84), d_1_–d_2_ 81 (80–82), d_2_–d_2_ 226 (224–228), c_1_–d_1_ 75 (74–78), c_3_–d_2_ 97 (95–99), e_1_–e_1_ 63 (61–65), e_1_–e_2_ 78 (74–79), e_2_–d_2_ 85 (83–86), e_2_–e_2_ 79 (75–79), f_1_–f_1_ 63 (60–65), f_2_–f_2_ 107 (105–108), f_1_–f_2_ 53 (50–54), f_1_–d_1_ 69 (66–70), h_1_–h_1_ 57 (55–59).

**Venter** (Fig. 2). Idiosoma ventrally with transverse striations from setae 1a to 3a; most of the area between 3a to 4a is transverse with few V-shaped striations laterally; transverse posterior to setae 4a; striations transverse regular anterior to aggenital setae (ag). The intercoxal setae 1a slightly longer than the distance 1a–1a. The intercoxal setae 3a just equal to distance 3a–3a. The intercoxal setae 4a 4/5 to the distance 4a–4a. Length of intercoxal and coxal setae: 1a 39 (35–40), 3a 52 (51–55), 4a 50 (48–52), 1b 54 (52–56), 1c 18 (16–20), 2b 37 (35–38), 2c 21 (20–24), 3b 23 (21–25), 4b 38 (36–39); aggenital setae ag 48 (44–48), ag–ag 27 (25–28); genital setae two pairs, g_1_ 32 (30–24), g_2_ 40 (38–42), g_1_–g_1_ 40 (39–44), g_2_–g_2_ 56 (52–57), g_1_–g_2_ 12 (10–14); anal setae three pairs, ps_1_ 21 (18–24), ps_2_ 37 (35–39), ps_3_ 58 (54–60), ps_1_–ps_1_ 33 (30–34), ps_2_–ps_2_ 26 (24–27), ps_3_–ps_3_ 19 (18–22); para-anal setae two pairs, h_2_ 33 (31–34), h_2–_ h_2_ 17 (16–18), h_3_ 38(35–40), h_3_–h_3_ 46 (45–48).

**Gnathosoma** (Figs 3–4). Stylophore elongate, slender and slightly notched anteriorly. Peritremes branched tube like compact anastomosing (Fig. 3). Scapular setae m 36 (34–37), m–m 32 (31–35). Palp femur and genu each with one seta, palp tibia with three setae, tibial claw strongly curved; palp tarsus with three setae, three eupathidia, one solenidion (Fig. 4).

**Legs** (Figs 5–8). Length of legs I–IV (without coxae) 336, 251, 276, 298 respectively. Leg I shorter than body length. Number of setae and solenidia (in parenthesis) on legs I–IV: coxae 2–2–1–1, trochanters 1–1–1–1, femora 5–5–3–3, genua 4–4–3–3, tibiae 9(1)–(8–9)–9–9; tarsi I with 12–14 tactile setae, two sets of duplex setae at distal end, two eupathidia and one/two solenidion; tarsi II with 8–9 tactile setae, one set of duplex setae, two eupathidia and one solenidion; tarsi III with 8–9 tactile setae and one solenidion; tarsi IV with 9 tactile setae and one solenidion. True claws pad like each with one pair of tenant hair; empodium pad–like with two rows of small tenant hairs.

**Male.** Not in collection.

##### Etymology.

The specific epithet is derived from the region “Arabia” from where type specimens were collected.

##### Type material.

Holotype and one paratype female, *Prosopis juliflora* (Fabaceae), Deesa Valley, Dessa, Tabuk, SA, 27°36.048'N, 036°25.592'E, October, 18, 2015, coll. J.H. Mirza.; seven paratype females, *Prosopis juliflora* (Fabaceae), Sharma, Near Red Sea, Tabuk, SA, 28°03.479'N, 035°17.186'E, October, 19, 2015, coll. M. Kamran.

##### Remarks.

The Paraplonobia (Anaplonobia) arabica sp. n. relates to Paraplonobia (Anaplonobia) prosopis (Tuttle & Baker, 1964), Paraplonobia (Anaplonobia) algarrobicola (Gonzalez, 1977) and Paraplonobia (Anaplonobia) boutelouae Tuttle & Baker, 1968 because of sharing following similar characters: dorsal body setae spatulate and distinctly shorter to the distances of setae next behind and widely spaced dorsal hysterosomal striations. Also, the new species closely resembles Paraplonobia (Anaplonobia) prosopis by setae c_1_ at least reaching half distance to the bases of setae d_1_. However, the new species differs from all related species by having stylophore anteriorly with slight incision (notch). The new species is also distinguished from Paraplonobia (Anaplonobia) prosopis by setae c_1_ reaching to the distance of setae d_1_ (2/3 vs.1/2), setae c_1_–c_1_ almost 1.5 times widely spaced than setae f_1_–f_1_ vs. almost sub/equally spaced in Paraplonobia (Anaplonobia) prosopis. The new species can be separated from other related species Paraplonobia (Anaplonobia) algarrobicola and Paraplonobia (Anaplonobia) boutelouae by the setae c_1_ reaching 2/3 to the distance of d_1_ vs. less than half as long as distances to the bases setae next behind in later species

#### Paraplonobia (Anaplonobia) haloxylonia sp. n.

Taxon classificationAnimaliaProstigmataTetranychidae

http://zoobank.org/09E8353-E635-4C38-B277-8D6DDC56C31A

Figs 9–10 , 11–15 , 16–19 , 20, 21 , 22–24 , 25–28

##### Diagnosis.

Dorsal setae lanceolate, densely serrate, not set on tubercles and distinctly shorter to the distances of setae next behind, dorsocentral setae (c1, d1 and e1) almost 1/3 to the distance of setae next behind, propodosoma medially with weak, longitudinal irregular striations, hysterosoma with transverse and closely spacedstriations medially, stylophore slightly notched anteriorly, peritremes anastomosed distally, with few long thread like branches, and hysterosomal striations closely spaced, leg I shorter than body.

##### Description of holotype female

(n = 39). Measurements of holotype followed by 38 paratypes (in parenthesis) (Figs 9–19).

**Figures 9, 10.**
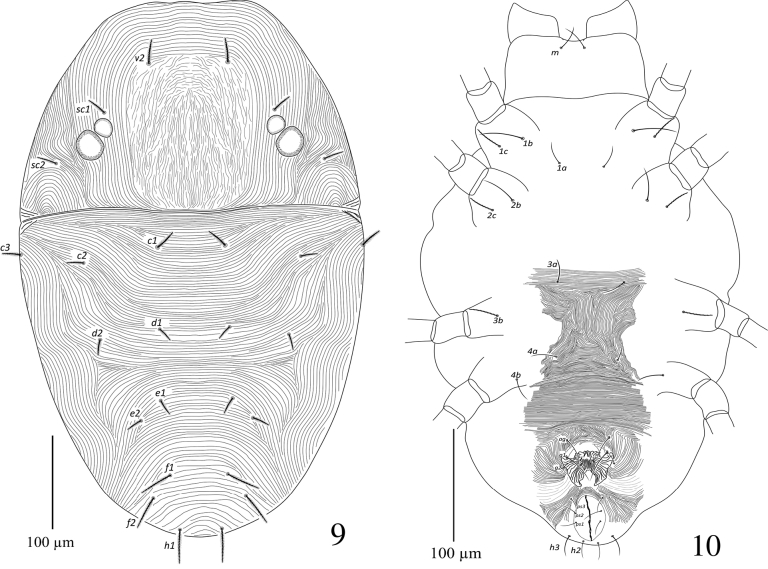
Paraplonobia (Anaplonobia) haloxylonia sp. n. adult female. **9** dorsum **10** venter.

**Figures 11–15.**
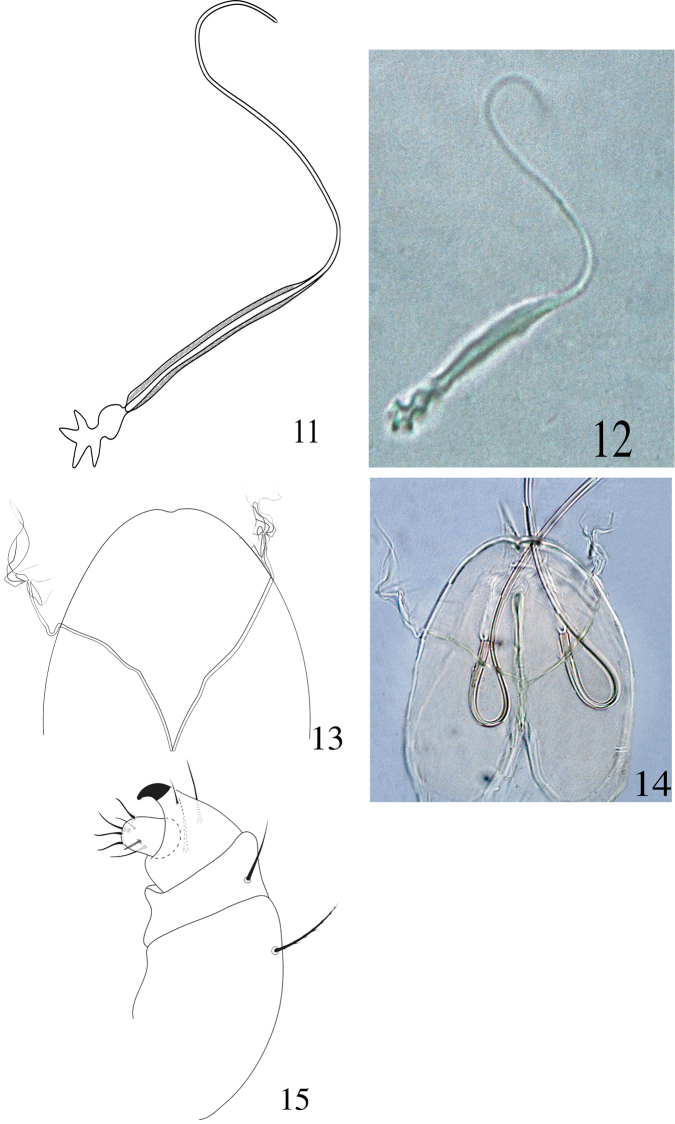
Paraplonobia (Anaplonobia) haloxylonia sp. n. adult female. **11, 12** spermatheca **13, 14** stylophore and peritremes **15** palp.

**Figures 16–19.**
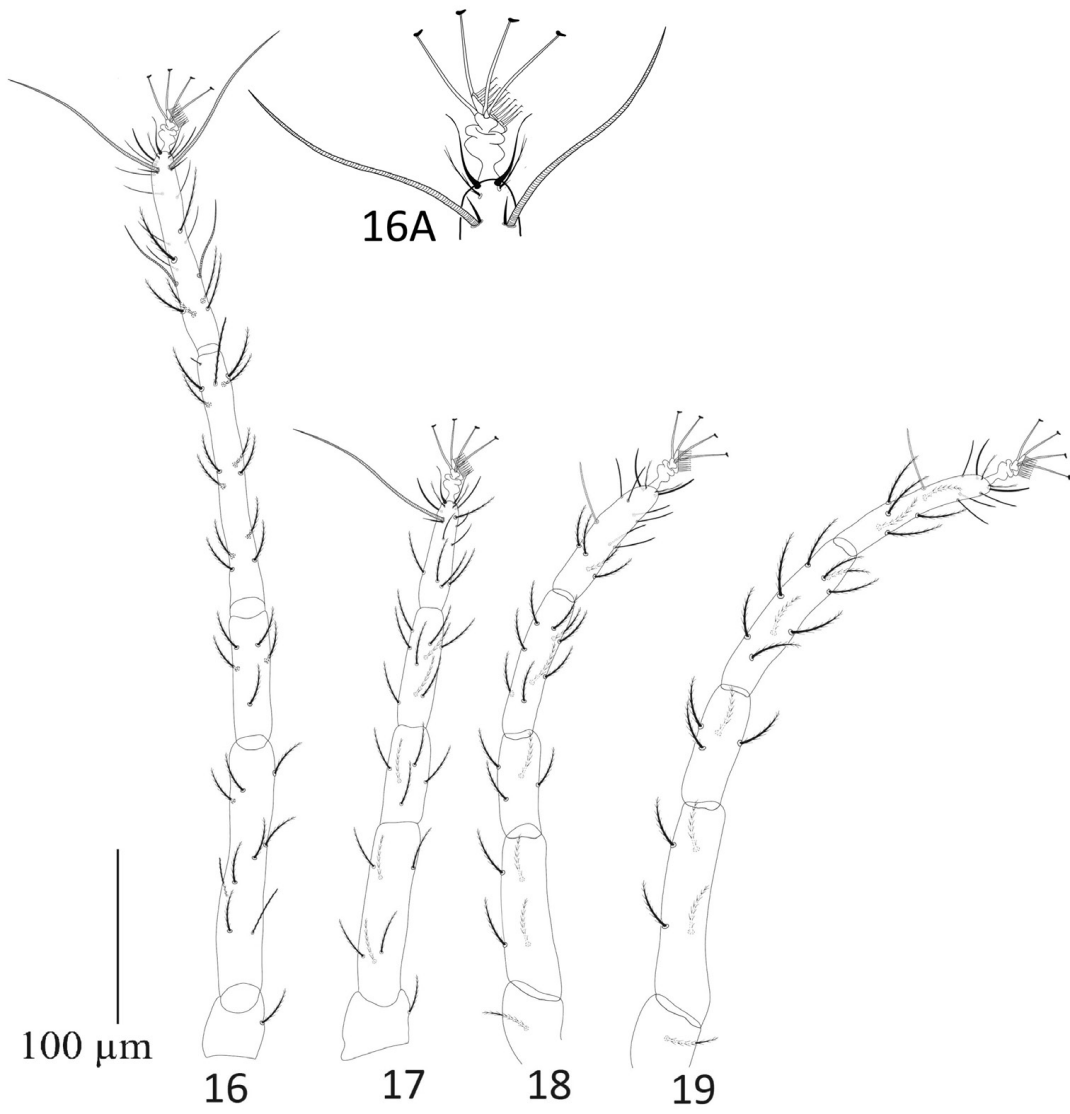
Paraplonobia (Anaplonobia) haloxylonia sp. n. **16** legI **16A** duplex setae, empodium and claws of tarsus I **17** leg II **18** leg III **19** leg IV.

**Dorsum** (Fig. 9). Body oval; length of idiosoma 583 (578–585), maximum width 372 (369–378), length of body (gnathosoma + idiosoma) 658 (655–663). Propodosoma medially with weak, longitudinal irregular striations; laterally longitudinal regular striations; hysterosomal striations medially transverse and closely spaced, laterally longitudinal irregular. Dorsal setae lanceolate, densely serrate, not present on tubercles and distinctly shorter to the distances of setae next behind, dorsocentral setae (c1, d1 and e1) almost 1/3 to the distance of setae next behind, Length of dorsal setae: v_2_ 28 (26–29), sc_1_ 24 (23–25), sc_2_ 22 (21–24), c_1_ 19 (18–21), c_2_ 22 (21–23), c_3_ 25 (24–28), d_1_ 15 (12–16), d_2_ 18 (17–19), e_1_ 16 (15–17), e_2_ 20 (19–20), f_1_ 25 (24–28), f_2_ 31 (29–32), h_1_ 34 (32–35). Distance between dorsal setae: v_2_–v_2_ 72 (70–73), v_2_–sc_1_ 75 (72–78), sc_1_–sc_2_ 66 (63–67), sc_1_–sc_1_ 167 (163–172), sc_2_–sc_2_ 254 (250–259), c_1_–c_1_ 90 (88–92), c_1_–c_2_ 75 (71–78), c_2_–c_3_ 81 (78–85), c_2_–c_2_ 231 (229–234), c_3_–c_3_ 373 (372–375), d_1_–d_1_ 91 (89–92), d_1_–d_2_ 65 (62–69), d_2_–d_2_ 204 (201–206), c_1_–d_1_ 103 (100–104), c_3_–d_2_ 160 (158–161), e_1_–e_1_ 55 (53–57), e_1_–e_2_ 53 (50–54), e_2_–d_2_ 85 (82–86), e_2_–e_2_ 150 (148–152), f_1_–f_1_ 60 (59–62), f_2_–f_2_ 80 (78–83), f_1_–f_2_ 28 (25–29), f_1_–d_1_ 93 (91–94), h_1_–h_1_ 31 (28–32).

**Venter** (Figs 10–12). Idiosoma ventrally with transverse simple striations from setae 1a to 3a; longitudinal irregular between setae 3a and 4a; transverse posterior to setae 4a; striations longitudinal irregular anterior to aggenital setae (ag). Length of intercoxal and coxal setae: 1a 25 (24–26), 3a 19 (19–21), 4a 22 (21–23), 1b 33 (31–33), 1c 22 (21–24), 2b 24(23–25), 2c 22 (21–23), 3b 23 (22–24), 4b 27 (26–28); aggenital setae, ag 28 (27–28), ag–ag 32 (29–32); genital setae two pairs, g_1_ 31 (30–33), g_2_ 20 (19–21), g_1_–g_1_ 32 (31–33), g_2_–g_2_ 35 (34–36), g_1_–g_2_ 10 (10–12); anal setae three pairs, ps_1_ 11 (10–12), ps_2_ 16 (15–17), ps_3_ 17(16–18), ps_1_–ps_1_ 16 (15–18), ps_2_–ps_2_ 22 (20–23), ps_3_–ps_3_ 26 (25–26); para–anal setae two pairs, h_2_ 16 (15–17), h_2–_ h_2_ 14 (13–16), h_3_ 17 (15–17), h_3_–h_3_ 31 (30–32) (Fig. 10). Spermathecae elongate, star shaped structure at distal end (Fig. 11–12).

**Gnathosoma** (Figs 13–15). Stylophore slightly notched anteriorly. Peritremes anastomosed distally, with few long thread like branches (Figs 13–14). Scapular setae m 22 (21–23), m–m 17 (16–18). Palp femur and genu each with one seta, palp tibia with three setae, tibial claw strongly curved; palp tarsus with three setae, three eupathidia, one solenidion (Fig. 15).

**Legs** (Figs 16–19). Length of legs I–IV (without coxae) 507, 328, 340, 400 respectively. Leg I shorter than body length. Number of setae and solenidia (in parenthesis) on legs I–IV: coxae 2–2–1–1, trochanters 1–1–1–1, femora 9–6–4–4, genua 5–5–4–4, tibiae 13(1)–9–9–9; tarsi I with 15 tactile setae, two sets of duplex setae at distal end, 11 tactile setae and two solenidia well proximal to duplex setae, two eupathidia; tarsi II with 10 tactile setae, one set of duplex setae, two eupathidia and one solenidion; tarsi III with 12 tactile setae and one solenidion; tarsi IV with 12 tactile setae and one solenidion. True claws pad like each with one pair of tenant hair; empodium pad-like with two rows of small tenant hairs.

**Figures 20, 21.**
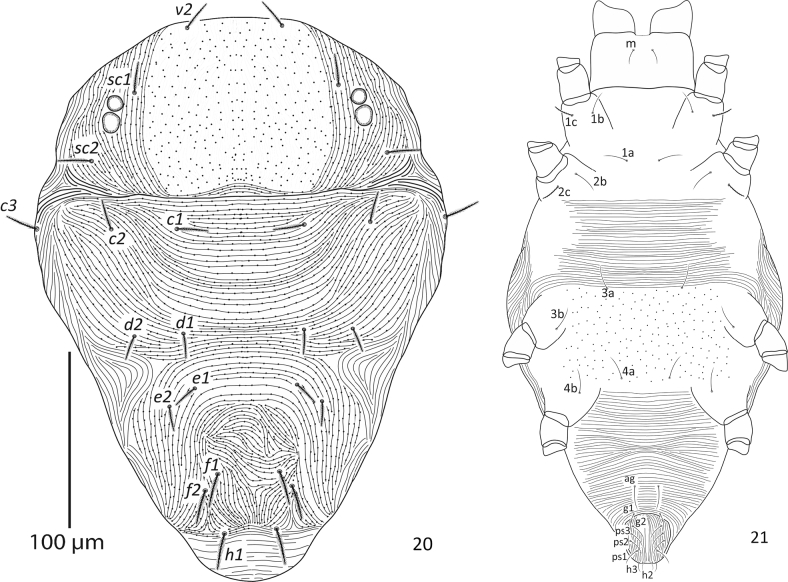
Paraplonobia (Anaplonobia) haloxylonia sp. n. adult male. **20** dorsum **21** venter.

**Figures 22–24.**
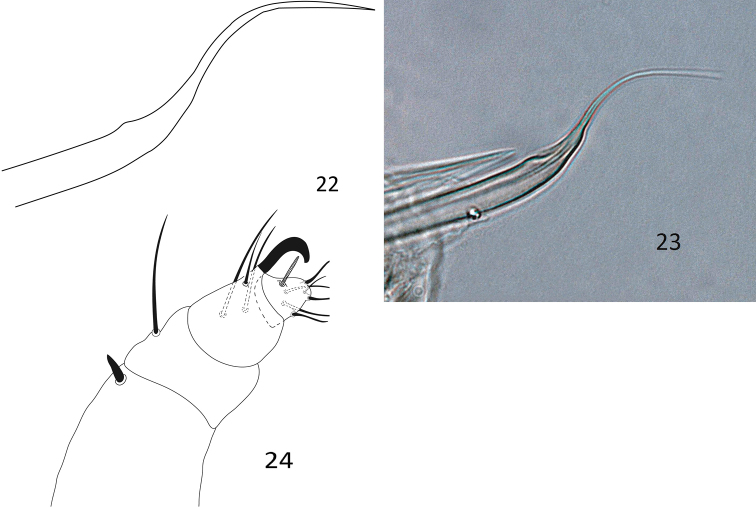
Paraplonobia (Anaplonobia) haloxylonia sp. n. adult male. **22–23** aedeagus **24** palp.

**Figures 25–28.**
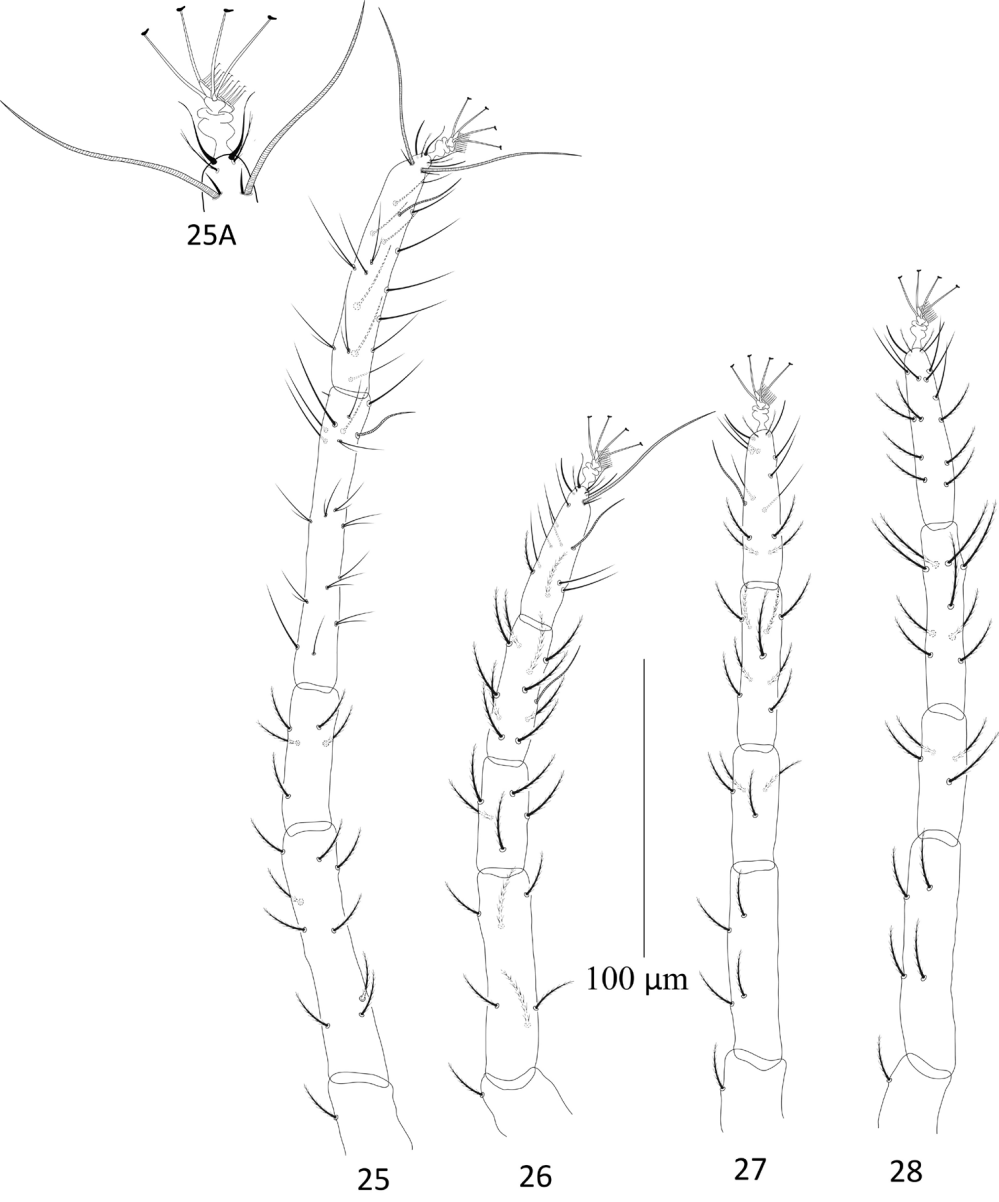
Paraplonobia (Anaplonobia) haloxylonia sp. n. adult male. **25** leg I **25A** duplex setae, empodium and claws of tarsus I **26** leg II **27** leg III **28** leg IV.

**Male (n = 11)** (Figs 20–28). **Dorsum** (Fig. 20). Body almost oval, slightly tapering caudally; idiosoma 320–325 long, 190 wide; striations on dorsum entirely dotted; propodosomal striations same as in female, hysterosomal also same as in female except longitudinal/oblique or irregular in the area medially between dorsal setae e1 and h1, shape of setae also same as in female.

**Venter** (Figs 21–23). Idiosoma ventrally with transverse striations except in the area between ventral setae 3a and 4a and genito-anal area; the area between 3a and 4a with simple dots (without striations); genito-anal setae five pair, genital setae two pairs (g_1_, g_2_), anal setae three pairs (ps_1_, ps_2_, ps_3_); para-anal setae two pairs (h_2_, h_3_); aedeagus up turned, broadly sigmoid, sharply tapering distally (Figs 22–23).

**Gnathosoma.** Stylophore and peritremes as in female; palp femur with small horn-like seta, palp genu with one dorsal seta, palp tibia with three setae and strongly curved tibial claw; palp tarsus thumb like with one solenidion, three eupathidia and three setae (Fig. 24).

**Legs** (Figs 25–28). Length of leg I–IV (without coxae) 366, 223, 250, 289 respectively. Setae with solenidion in parenthesis on legs I–IV as; coxae 2–2–1–1, trochanters 1–1–1–1, femora 9–6–4–4, genua 5–5–4–4, tibiae 9(2)+8duplex–10(1)–9–9, tarsus I with six pairs of duplex setae (two pairs distally, two pairs at mid and two pairs at proximal part of the tarsus), 15 tactile setae, two eupathidia, one solenidion, tarsus II with one duplex seta, nine tactile setae, two eupathidia, one solenidion, tarsus III with 12 tactile setae, one solenidion, tarsus IV with 13 tactile setae, one solenidion. True claws pad like each with one pair of tenant hair; empodium pad-like with two rows of small tenant hairs.

##### Etymology.

The specific epithet is derived after the host plant, *Haloxylon salicornicum* from which some type specimens were collected.

##### Type material.

Holotype female, one male and two female paratypes, *Haloxylon salicornicum* (Amaranthaceae), Salbookh Road, Dariyah, Riyadh, SA, 24°30.649'N, 46°46.615'E, September, 18, 2012, coll. M. Kamran; four males and 22 female paratypes, *Hilaria* sp. (Poaceae), Tashlia, Heyer Road, Riyadh, SA, 24°29.000'N, 46°47.890'E, January, 17, 2015, coll. J.H. Mirza; five males and four females paratypes, *Hilaria* spp. (Poaceae), Sanabal Farm, Kharaj, Riyadh, SA, 24°16.999'N, 47°11.854'E, January, 23, 2015, coll. M. Kamran.

**Remarks.** The Paraplonobia (Anaplonobia) haloxylonia sp. n. closely resembles Paraplonobia (Anaplonobia) contiguus (Chaudhri, Akbar and Rasool 1974) because both species sharing the following set of similar characters; peritremes distally with few branches, dorsal body setae short, subequal in length, lanceolate, prodorsal shield entirely with longitudinal striaitons and hysterosomal striations closely spaced. The new species, differs from Paraplonobia (Anaplonobia) contiguus by comparative length of leg I (shorter than body vs. longer than body), dorsocentral setae (c1, d1 and e1) almost 1/3 to the distance of setae next behind vs. more than half, number of setae on genu I (5 vs. 4) in Paraplonobia (Anaplonobia) contiguus.

#### Paraplonobia (Anaplonobia) tabukensis sp. n.

Taxon classificationAnimaliaProstigmataTetranychidae

http://zoobank.org/57BF2D3A-80B0-4C7E-90CD-FACB4543B5FF

Figs 29–30 , 31, 32 , 33–36

##### Diagnosis.

Dorsal setae slightly lanceolate, densely serrate, not present on tubercles and distinctly shorter to the distances of setae next behind, prodorsum entirely with longitudinal striaitons, hysterosomal striations closely spaced, peritremes complex anastomosed distally, stylophore slightly rounded anteriorly, leg I shorter than body length, number of setae on femur I–IV 8–6–3–3, number of setae on genu I–IV 4–5–3–3.

##### Description of holotype female

(n = 3). Measurements of holotype followed by 2 paratypes (in parenthesis) (Figs 29–36).

**Figures 29, 30.**
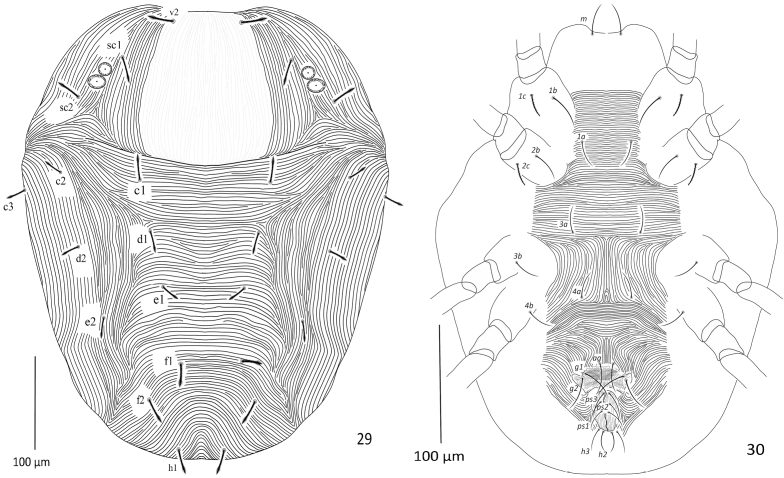
Paraplonobia (Anaplonobia) tabukensis sp. n. adult female **29** dorsum **30** venter.

**Figures 31, 32.**
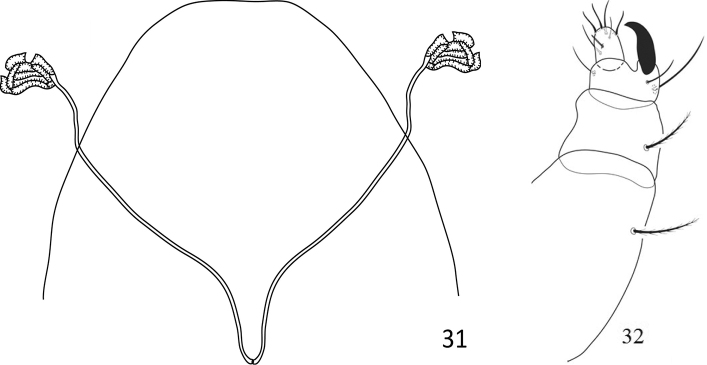
Paraplonobia (Anaplonobia) tabukensis sp. n. adult female. **31** Stylophore and peritremes **32** palp.

**Figures 33–36.**
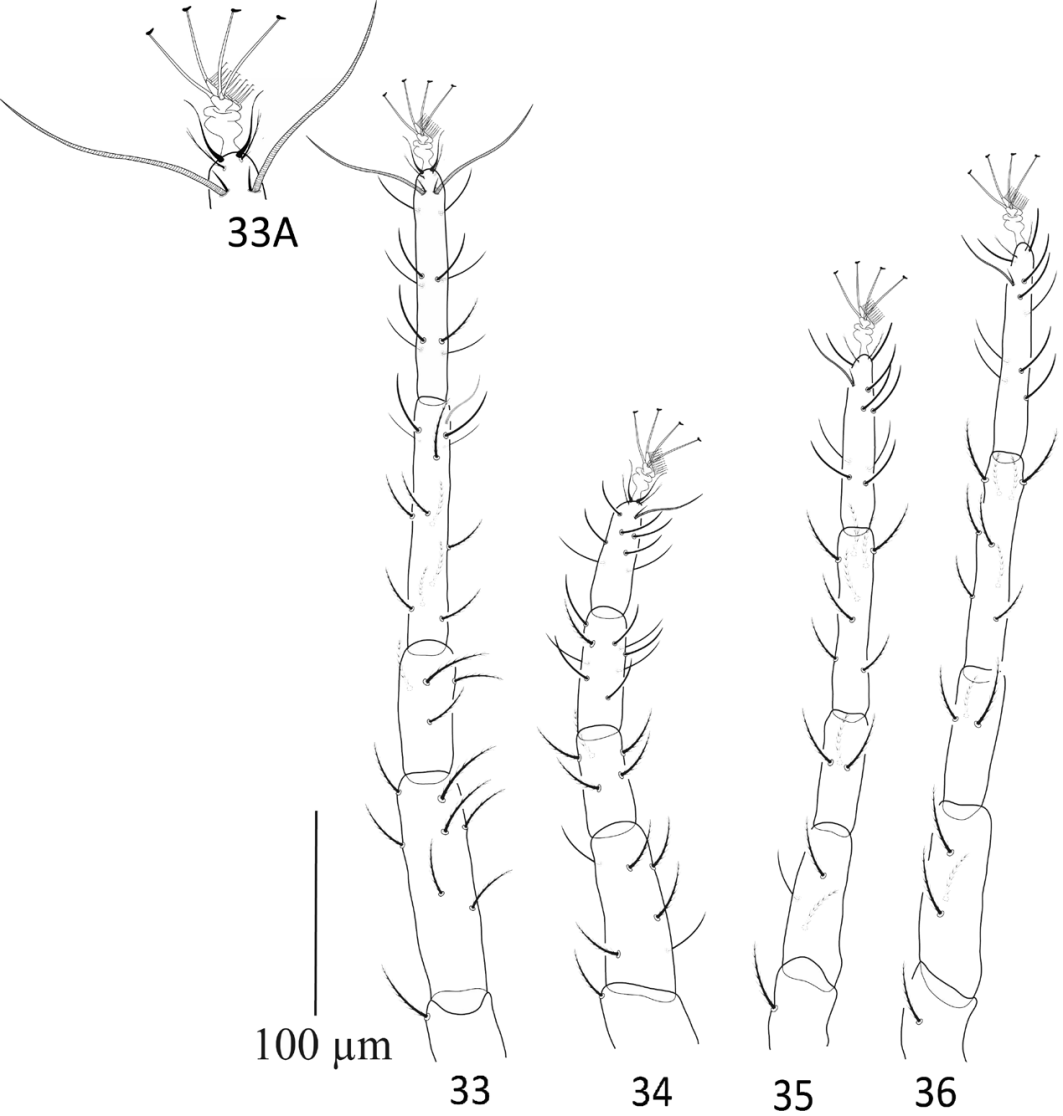
Paraplonobia (Anaplonobia) tabukensis sp. n. **33** leg I **33A** duplex setae, empodium and claws of tarsus I **34** leg II **35** leg III **36** leg IV.

**Dorsum** (Fig. 29). Body rounded; length of idiosoma 483 (480–490), maximum width 445 (440–450), length of body (gnathosoma + idiosoma) 595 (590–610). Propodosoma medially with weak and laterally with strong longitudinal regular striations; hysterosomal striations medially transverse and closely spaced, laterally longitudinal irregular. Dorsal setae slightly lanceolate, densely serrate, not present on tubercles and and distinctly shorter to the distances of setae next behind, dorsocentral setae (c1, d1 and e1) almost 1/3 to the distance of setae next behind. Length of dorsal setae: v_2_ 34 (32–36), sc_1_ 29 (28–31), sc_2_ 30 (28–32), c_1_ 28 (26–30), c_2_ 26 (24–28), c_3_ 29 (28–32), d_1_ 23 (21–25), d_2_ 22 (21–24), e_1_ 21 (20–23), e_2_ 22 (21–24), f_1_ 23 (21–24), f_2_ 26 (24–27), h_1_ 27 (25–29). Distance between dorsal setae: v_2_–v_2_ 89 (85–90), v_2_–sc_1_ 68 (65–690), sc_1_–sc_2_ 68 (67–70), sc_1_–sc_1_ 204 (202–206), sc_2_–sc_2_ 301 298–302), c_1_–c_1_ 138 (135–140), c_1_–c_2_ 91 (89–92), c_2_–c_3_ 79 (75–80), c_2_–c_2_ 327 (325–328), c_3_–c_3_ 424 (422–426), d_1_–d_1_ 119 (118–120), d_1_–d_2_ 91 (89–92), d_2_–d_2_ 295 (292–298), c_1_–d_1_ 88 (86–89), c_3_–d_2_ 110 (109–112), e_1_–e_1_ 27 (25–28), e_1_–e_2_ 85 (84–86), e_2_–d_2_ 85 (84–86), e_2_–e_2_ 229 (228–231), f_1_–f_1_ 78 (76–80), f_2_–f_2_ 113 (110–114), f_1_–f_2_ 35 (33–36), f_1_–d_1_ 82 (81–84), h_1_–h_1_ 53 (52–56).

**Venter** (Fig. 30). Idiosoma ventrally with transverse simple striations from setae 1a to 3a; longitudinal regular between setae 3a and 4a; transverse posterior to setae 4a; striations longitudinal regular anterior to aggenital setae (ag). Length of intercoxal and coxal setae: 1a 40 (38–42), 3a 32 (31–34), 4a 32 (30–35), 1b 46 (44–47), 1c 32 (31–34), 2b 30 (29–34), 2c 29 (28–31), 3b 32 (31–34), 4b 32 (31–35); aggenital setae (ag) 42 (41–45), ag–ag 23 (21–25); genital setae two pairs, g_1_ 43 (40–44), g_2_ 39 (35–40), g_1_–g_1_ 52 (50–55), g_2_–g_2_ 60 (58–64), g_1_–g_2_ 12 (10–13); anal setae three pairs, ps_1_ 20 (18–21), ps_2_ 26 (24–27), ps_3_ 28 (27–29), ps_1_–ps_1_ 23 (20–24), ps_2_–ps_2_ 32 (31–35), ps_3_–ps_3_ 23 (21–26); para-anal setae two pairs, h_2_ 27 (26–28), h_2–_ h_2_ 11 (10–13), h_3_ 32 (31–34), h_3_–h_3_ 28 (27–29).

**Gnathosoma** (Figs 31–32). Stylophore rounded anteriorly. Peritremes small compact anastomosed distally (Fig. 31). Scapular setae m 28 (27–29), m–m 37 (26–39). Palp femur and genu each with one seta, palp tibia with three setae, tibial claw strongly curved; palp tarsus with three setae, three eupathidia, one solenidion (Fig. 32).

**Legs** (Figs 33–36). Length of legs I–IV (without coxae) 450, 282, 345, 408 respectively. Leg I shorter than body length. Number of setae and solenidia (in parenthesis) on legs I–IV: coxae 2–2–1–1, trochanters 1–1–1–1, femora 8–6–3–3, genua 4–5–3–3, tibiae 13(1)–9–8–8; tarsi I with 10 tactile setae, two sets of duplex setae at distal end, all tactile setae well proximal to duplex setae, two eupathidia; tarsi II with 7 tactile setae, one set of duplex setae, two eupathidia; tarsi III with 11 tactile setae, one set of duplex setae,; tarsi IV with 11 tactile setae one set of duplex setae,. True claws pad like each with one pair of tenant hair; empodium pad-like with two rows of small tenant hairs.

**Male.** Not in collection.

##### Etymology.

The specific epithet is derived from the region of Saudi Arabia, Tabuk, from where it was collected.

##### Type material.

Holotype female, two paratype females, *Haloxylon salicornicum* (Amaranthaceae), 30 km Tabuk road, Sharma, Tabuk region, SA, 28°03.479'N, 035°17.186'E, October, 19, 2015, coll. M. Kamran and J.H. Mirza.

##### Remarks.

The Paraplonobia (Anaplonobia) tabukensis sp. n. closely resembles Paraplonobia (Anaplonobia) theroni (Meyer 1974) because both species share the following set of similar characters; dorsal body setae, lanceolate and distinctly shorter to the distances of setae next behind, prodorsum entirely with longitudinal striaitons, hysterosomal striations closely spaced, peritremes complex anastomosed distally (Meyer 1974, 1987). The new species differs from Paraplonobia (Anaplonobia) theroni by shape of stylophore anteriorly (rounded vs. slightly indented), number of setae on femur I–IV (8–6–3–3 vs. 9–6–4–4), number of setae on genu I–IV (4–5–3–3 vs. 5–5–6–6), number of setae on tibia III (8 vs. 6) and on tarsi I–II excluding duplex setae and solenidia (10–7 vs. 18–14) in Paraplonobia (Anaplonobia) theroni.

#### Key to the world species of the genus *Paraplonobia* (Prostigmata: Tetranychidae) (after Meyer 1987).

**Table:** 

1	Coxal formula not exceeding 3–3–1–1	**2**
–	Coxal formula 4–3–2–2, dorsal body setae serrate pointed at the tip not set on tubercles, peritremes simple, empodial pad and true claws equal in length	**subg. *Brachynychus*, species Paraplonobia (Brachynychus) cousiniae (Mitrofanov & Strunk.)**
2	Peritremes anastomosed	**subg. *Anaplonobia*, 11**
–	Peritremes simple	**subg. *Paraplonobia*, 3**
3	Stylophore rounded anteriorly	**4**
–	Stylophore notched anteriorly	**5**
4	Dorsal body setae slightly lanceolate, leg I shorter that body	**Paraplonobia (Paraplonobia) edenvillensis Meyer**
–	Dorsal body setae slender, leg I about as long as body	**Paraplonobia (Paraplonobia) myops (Pritchard & Baker)**
5	Dorsal body setae generally slender or slightly lanceolate and pointed distally	**6**
–	Dorsal body setae broadly lanceolate	**9**
6	First three pair of dorsocentral setae c_1_, d_1_ and e_1_ about half as long as distance between bases of consecutive setae	**7**
–	First three pair of dorsocentral setae c_1_, d_1_ and e_1_ minute about a third to a fourth as long as the distance between bases of consecutive setae	**8**
7	Length of body 466 µm (530 µm including gnathosoma), leg I as long as body, posterior opisthosomal setae longer than longitudinal distance between their bases	**Paraplonobia (Paraplonobia) hilariae Tuttle & Baker**
–	Length of body 380 µm, leg I 160 µm long, shorter than body, posterior opisthosomal setae shorter than longitudinal distance between their bases	**Paraplonobia (Paraplonobia) herniariae (Bagdasarian)**
8	Body elongate, length of body 345 µm, length of leg I 191 µm (without coxa and trochanter)	**Paraplonobia (Paraplonobia) boutelouae Baker & Tuttle**
–	Body oval, length of body 570 µm, length of leg I 419 µm (without coxa and trochanter)	**Paraplonobia (Paraplonobia) dactyloni Smiley & Baker**
9	Dorsocentral setae (c1, d1, e1 and f1) more than half as long as distances between consecutive setae, leg I shorter than body	**10**
–	Dorsocentral setae (c1, d1, e1 and f1) almost half as long as distances between consecutive setae, leg I shorter than body	**Paraplonobia (Paraplonobia) tridens Tuttle & Baker**
10	Peritremes terminating in a ball-like rounded structure; prodorsum with a wellmarked punctate shield; tibia IV with 8 setae	**Paraplonobia (Paraplonobia) penicillatus Chaudhri et al.**
–	Peritremes terminating in oval shaped structure; prodorsum without a well-marked punctate shield; tibia IV with 7 setae	**Paraplonobia (Paraplonobia) echinopsili (Wainstein)**
11	Dorsal body setae slightly shorter/as long as/ longer than distances between their bases	**28**
–	Dorsal setae distinctly shorter than distances between their bases	**12**
12	Dorsal integument striated, without tubercles or lumps	**13**
–	Dorsal integument provided with tubercles or lumps forming a distinct pattern along with striation	**Paraplonobia (Anaplonobia) glebulenta (Meyer)**
13	Dorsal body setae slender, setiform	**14**
–	Dorsal body setae broadly spatulate, subspatulate or lanceolate	**15**
14	Stylophore indented anteriorly, dorsocentral setae c_1_, d_1_ and e_1_ about 2/3 of the distance between their basis, peritremes weakly anastomosed	**Paraplonobia (Anaplonobia) inornata (Meyer)**
–	Stylophore rounded anteriorly, dorsocentral setae c_1_, d_1_ and e_1_ about half the distance between, peritremes strongly anastomosed, stylophore rounded anteriorly.	**Paraplonobia (Anaplonobia) ambrosiae (Tuttle et al.)**
15	All dorsal body setae spatulate, subspatulate, expanded distally	**16**
–	Most of dorsal body setae lanceolate, not expanded distally	**19**
16	First pair of dorsocentral setae c_1_ less than half as long as distances to the bases setae next behind	**17**
–	First pair of dorsocentral setae c1 at least reaching 1/2 or 2/3 of distance to the bases of setae next behind	**18**
17	Prodorsum medially with irregular broken striations	**Paraplonobia (Anaplonobia) boutelouae Tuttle & Baker**
–	Prodorsum medially with regular longitudinal striations	**Paraplonobia (Anaplonobia) algarrobicola (Gonzalez)**
18	First pair of dorsocentral setae c1 reaching one half to the distance of setae next behind, setae c1 and f1 almost sub/equally spaced	**Paraplonobia (Anaplonobia) prosopis (Tuttle & Baker)**
–	First pair of dorsocentral setae c1 reaching 2/3 to the distance of setae next behind, setae c1 almost 1.5 times widely spaced than setae f1	**Paraplonobia (Anaplonobia) arabica sp. n.**
19	Hysterosomal setae d1and e1 lanceolate and about half as long as f1, setae f1 spatulate	**Paraplonobia (Anaplonobia) brickellia Baker & Tuttle**
–	Dorsocentral setae subequal in length, lanceolate serrate	**20**
20	Prodorsum entirely with longitudinal striations	**21**
–	Median area of prodorsum entirely/partially with transverse striations	**26**
21	Peritremes ending with few irregular branches	**22**
–	Peritremes distally with complex anastomosed	**24**
22	Stylophore slightly indented anteriorly, dorsum with closely spaced striations	**23**
–	Stylophore rounded anteriorly, dorsum with widely spaced striations	**Paraplonobia (Anaplonobia) acharis (Pritchard & Baker)**
23	Leg I distinctly longer than the body, first pair of dorsocentral setae c1 more than half to the distance of setae next behind	**Paraplonobia (Anaplonobia) contiguus (Chaudhri et al.)**
–	Leg I shorter than body, first pair dorsocentral setae c1 1/3 to the distance of setae next behind	**Paraplonobia (Anaplonobia) haloxylonia sp. n.**
24	Dorsum with widely spaced striaitons, femora I with 11 setae	**Paraplonobia (Anaplonobia) candicans (Meyer)**
–	Dorsum with closely spaced striations, femora I with 8 or 9 setae	**25**
25	Stylophore rounded anteriorly, setae on femora I–IV 8–6–3–3, setae of genua I–IV 4–5–3–3	**Paraplonobia (Anaplonobia) tabukensis sp. n.**
–	Stylophore indented anteriorly, setae on femora I–IV 9–6–4–4, setae of genua I–IV 5–5–6–6	**Paraplonobia (Anaplonobia) theroni (Meyer)**
26	Propodosomal shield medially with two distinct bands of transverse striations	**Paraplonobia (Anaplonobia) daryaensis Chaudhri et al.**
–	Propodosomal shield entirely with transverse strations	**27**
27	Leg I shorter than body, peritremes weakly anastomosed	**Paraplonobia (Anaplonobia) harteni (Meyer)**
–	Leg I longer than body, peritremes with complex anastomose	**Paraplonobia (Anaplonobia) concolor Chaudhri et al.**
28	Stylophore anteriorly rounded	**29**
–	Stylophore anteriorly deeply notched	**Paraplonobia (Anaplonobia) tshipensis (Meyer)**
29	Dorsal body setae slender/setifrom	**31**
–	Dorsal body setae spatulate/subspatulate	**30**
30	Dorsal body setae set on tubercles, longer than the distances of setae next behind, propodosoma with broken striations	**Paraplonobia (Anaplonobia) juliflorae (Tuttle & Baker)**
–	Dorsal body setae not set on tubercles, as long as or slightly shorter to the distances of setae next behind, propodosoma medially with basket weaved pattern	**Paraplonobia (Anaplonobia) euphorbiae (Tuttle & Baker)**
31	Opisthosomal setae much longer than the distance to the setae next in line	**Paraplonobia (Anaplonobia) coldeniae (Tuttle & Baker)**
–	Opisthosomal setae as long as the distance to the setae next in line	**32**
32	Prodorsal shield pebbled, most of opisthosomal setae set on tubercles	**Paraplonobia (Anaplonobia) calame (Pritchard & Baker)**
–	Prodorsal shield tuberculate/striate, opisthosomal setae not set on tubercles	**33**
33	Opisthosomal striations closely spaced with fine lobes	**Paraplonobia (Anaplonobia) artemisia Baker & Tuttle**
–	Prodorsal shield tuberculate	34
34	Opisthosomal striations mostly broad folds and covered with tubercles, peritreme small bulb like anastomosing	**Paraplonobia (Anaplonobia) berberis Baker & Tuttle**
–	Opisthomosal striations comparatively closely spaced with fine lobes, peritremes elongate anastomose	**Paraplonobia (Anaplonobia) allionia Baker & Tuttle**

#### Genus Neopetrobia

Taxon classificationAnimaliaProstigmataTetranychidae

Wainstein, 1956

Monoceronychus : Pritchard and Baker 1955: 77.
Neopetrobia : Wainstein 1956: 151, Wainstein 1960a: 128, Tuttle and Baker 1968: 57, Meyer 1974: 93–94.

##### Type species.

*Neopetrobia dubinini* Wainstein, 1956.

##### Diagnosis.

Based on Baker and Tuttle 1968, Gutierrez 1955, Meyer 1974, Meyer 1987, and Bolland et al. 1998.

True claws pad like, each bearing a pair of tenant hairs; empodial pad longer than true claws, bearing a row of tenant hairs, distally not coalescent; dorsum with 3 pairs of prodorsal setae which are short and spindle shaped or spatulate; setal tubercles small or nonexistent; fourth pair of dorsocentral setae (f_1_) widely spaced, not normal as c_1_; peritremes anastomosing distally.

#### Subgenus Neopetrobia

Taxon classificationAnimaliaProstigmataTetranychidae

Wainstein

##### Diagnosis.

Based on Gutierrez 1985, and Bolland et al. 1998.

Integument without tuberculate or reticulate pattern; dorsal setae rounded or spindle-shaped.

#### Neopetrobia mcgregori

Taxon classificationAnimaliaProstigmataTetranychidae

(Pritchard & Baker)

Figs 37–38 , 39, 40 , 41–44

Monoceronychus mcgregori Pritchard & Baker, 1955.
Neopetrobia mcgregori (Pritchard & Baker) Meyer, 1987. Bolland et al. 1998.

##### Redescription.

**Female** (n=9). Body oval; length of idiosoma 369–372, maximum width 238–241, length of body (gnathosoma + idiosoma) 430–433.

**Figures 37, 38.**
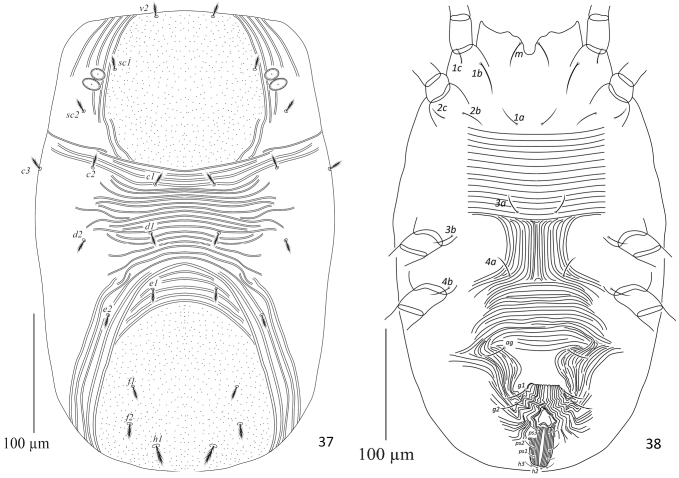
Neopetrobia (Neopetrobia) mcgregori (Pritchard & Baker) adult female. **37** dorsum **38** venter.

**Dorsum** (Fig. 37). Propodosoma without anterior projections. Dorsum of opisthosoma and most of opisthosoma with nearly smooth integument, metapodosomal dorsum with widely spaced strong striations. Dorsal body setae minute, lanceolate, densely serrate, not present on tubercles. Length of dorsal setae: v_2_ 13–14, sc_1_ 14–15, sc_2_ 13–14, c_1_ 13–14, c_2_ 12–13, c_3_ 10–11, d_1_ 11–12, d_2_ 12–13, e_1_ 10–11, e_2_ 12–13, f_1_ 11–12, f_2_ 15–16, h_1_ 16–17. Distance between dorsal setae: v_2_–v_2_ 54–56, v_2_–sc_1_ 48–50, sc_1_–sc_2_ 47–50, sc_1_–sc_1_ 113–114, sc_2_–sc_2_ 165–167, c_1_–c_1_ 57–58, c_1_–c_2_ 50–52, c_2_–c_3_ 41–42, c_2_–c_2_ 161–162, c_3_–c_3_ 234–236, d_1_–d_1_ 57–58, d_1_–d_2_ 56–57, d_2_–d_2_ 160–161, c_1_–d_1_ 57–58, c_3_–d_2_ 79–80, e_1_–e_1_ 54–56, e_1_–e_2_ 45–47, e_2_–d_2_ 64–66, e_2_–e_2_ 135–136, f_1_–f_1_ 80–82, f_2_–f_2_ 86–88, f_1_–f_2_ 31–32, f_1_–d_1_ 79–80, h_1_–h_1_ 38–40.

**Venter** (Fig. 38). Idiosoma ventrally with transverse simple widely spaced striations from setae 1a to 3a; longitudinal regular between setae 3a and 4a; transverse posterior to setae 4a; striations transverse regular anterior to aggenital setae (ag). Length of intercoxal and coxal setae: 1a 18–19, 3a 19–20, 4a 15–16, 1b 30–31, 1c 13–14, 2b 16–17, 2c 10–13, 3b 15–17, 4b 11–12; aggenital setae (ag) 26–27, ag–ag 38–39; genital setae two pairs, g_1_ 17–18, g_2_ 21–22, g_1_–g_1_ 41–42, g_2_–g_2_ 76–78, g_1_–g_2_ 21–22; anal setae three pairs, ps_1_ 11–12, ps_2_ 10–11, ps_3_ 12–13, ps_1_–ps_1_ 11–13, ps_2_–ps_2_ 16–18, ps_3_–ps_3_ 11–13; para-anal setae two pairs, h_2_ 11–13, h_2–_ h_2_ 7–9, h_3_ 7–8, h_3_–h_3_ 17–19.

**Figures 39, 40.**
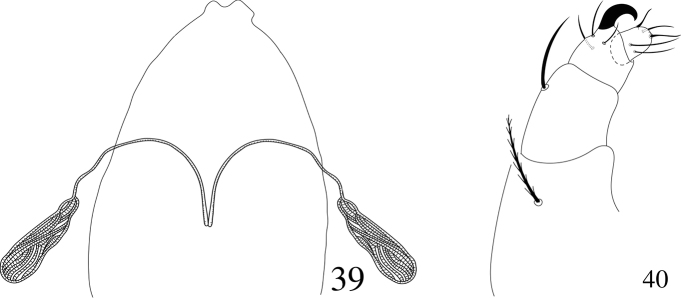
Neopetrobia (Neopetrobia) mcgregori (Pritchard & Baker) adult female. **39** stylophore and peritremes **40** palp.

**Gnathosoma** (Figs 39–40). Stylophore slender, the sides angularly converging anteriorly and with a small mediocephalic emargination. Peritremes anastomosing with distal enlargement slender. Scapular setae m 17–18, m–m 19–21. Palp femur and genu each with one seta, palp tibia with three setae, tibial claw strongly curved; palp tarsus with two setae, two eupathidia, one solenidion.

**Figures 41–44.**
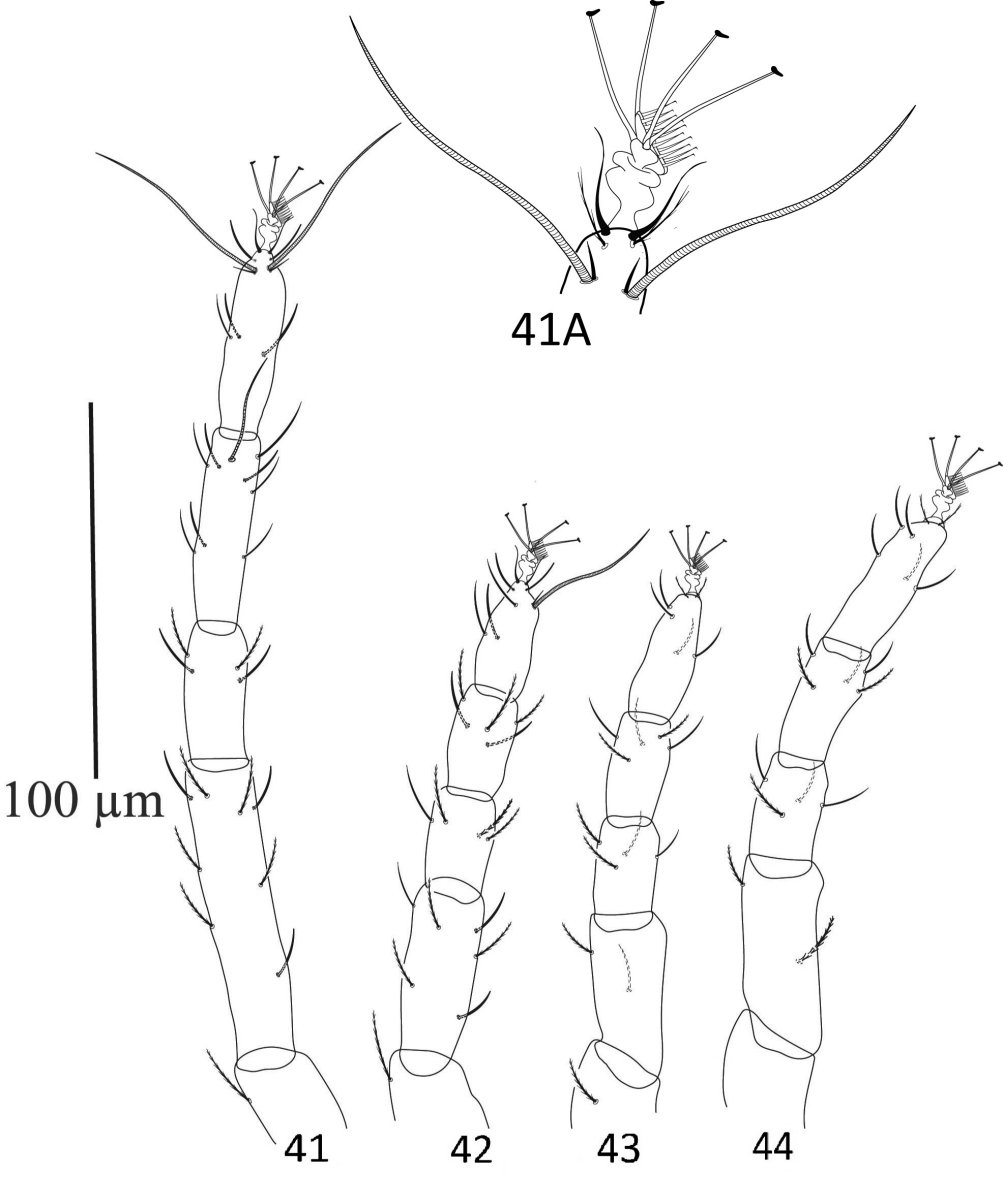
Neopetrobia (Neopetrobia) mcgregori (Pritchard and Baker) adult female. **41** leg I **41A** duplex setae, empodium and claws of tarsus I **42** leg II **43** leg III **44** leg IV.

**Legs** (Fig. 41–44). Length of legs I–IV (without coxae) 240, 150, 148, 180 respectively. Number of setae and solenidia (in parenthesis) on legs I–IV: coxae 2–2–1–1, trochanters 1–1–1–0, femora 8–6–2–2, genua 4–4–4–4, tibia 8(1)–9–9–9; tarsi I with 11 tactile setae, two sets of duplex setae at distal end, three setae proximal to duplex setae, two eupathidia and one solenidion; tarsi II with nine tactile setae, one set of duplex setae, two setae proximal to duplex setae, one setae in line with duplex setae, two eupathidia and one solenidion; tarsi III with six tactile setae; tarsi IV with seven tactile setae. True claws pad like each with one pair of tenant hair; empodium pad-like with two rows of small tenant hairs.

##### Materials examined.

12 females, *Cynodon dactylon* (Poaceae), near exit10, King Abdullah Road, Riyadh, SA, 24°45.826'N, 46°45.470'E, September 07, 2015, coll. M. Kamran and E. M. Khan.

##### Remarks.

*Neopetrobia mcgregori* was originally described very briefly under the genus *Monoceronychus* and has been only reported from Miami shores of Florida, USA (Pritchard and Baker 1955). Later, it was moved to the genus *Neopetrobia* on the basis of widely spaced fourth pair of dorsocentral setae (f_1_) (Bolland et al. 1998). Worldwide, this is the second report of this species and no obvious differences have been observed in Saudi Arabian specimens from the original description.

## Supplementary Material

XML Treatment for Paraplonobia

XML Treatment for Anaplonobia

XML Treatment for Paraplonobia (Anaplonobia) arabica

XML Treatment for Paraplonobia (Anaplonobia) haloxylonia

XML Treatment for Paraplonobia (Anaplonobia) tabukensis

XML Treatment for Neopetrobia

XML Treatment for Neopetrobia

XML Treatment for Neopetrobia mcgregori
